# Postural control strategy after incomplete spinal cord injury: effect of sensory inputs on trunk–leg movement coordination

**DOI:** 10.1186/s12984-020-00775-2

**Published:** 2020-10-27

**Authors:** Alireza Noamani, Jean-François Lemay, Kristin E. Musselman, Hossein Rouhani

**Affiliations:** 1 Department of Mechanical Engineering, University of Alberta, 10-368 Donadeo Innovation Centre for Engineering, 9211-116 Street NW, Edmonton, AB T6G 1H9 Canada; 2 CIUSSS du Centre-Sud-de-L’Île-de-Montréal (Installation Gingras-Lindsay), Montreal, QC Canada; 3 School of Rehabilitation, Université de Montréal, Montreal, QC Canada; 4 SCI Mobility Lab, KITE, Toronto Rehabilitation Institute-University Health Network, Toronto, ON Canada; 5 Department of Physical Therapy, Faculty of Medicine, University of Toronto, Toronto, ON Canada

**Keywords:** Spinal cord injury, Multi-joint coordination, Ankle strategy, Hip strategy, Coherence, Inertial measurement unit

## Abstract

**Background:**

Postural control is affected after incomplete spinal cord injury (iSCI) due to sensory and motor impairments. Any alteration in the availability of sensory information can challenge postural stability in this population and may lead to a variety of adaptive movement coordination patterns. Hence, identifying the underlying impairments and changes to movement coordination patterns is necessary for effective rehabilitation post-iSCI. This study aims to compare the postural control strategy between iSCI and able-bodied populations by quantifying the trunk–leg movement coordination under conditions that affects sensory information.

**Methods:**

13 individuals with iSCI and 14 aged-matched able-bodied individuals performed quiet standing on hard and foam surfaces with eyes open and closed. We used mean Magnitude-Squared Coherence between trunk–leg accelerations measured by accelerometers placed over the sacrum and tibia.

**Results:**

We observed a similar ankle strategy at lower frequencies (f ≤ 1.0 Hz) between populations. However, we observed a decreased ability post-iSCI in adapting inter-segment coordination changing from ankle strategy to ankle–hip strategy at higher frequencies (f > 1.0 Hz). Moreover, utilizing the ankle–hip strategy at higher frequencies was challenged when somatosensory input was distorted, whereas depriving visual information did not affect balance strategy.

**Conclusion:**

Trunk–leg movement coordination assessment showed sensitivity, discriminatory ability, and excellent test–retest reliability to identify changes in balance control strategy post-iSCI and due to altered sensory inputs. Trunk–leg movement coordination assessment using wearable sensors can be used for objective outcome evaluation of rehabilitative interventions on postural control post-iSCI.

## Background

Regaining walking function and maintaining a steady standing posture are listed as top priorities for individuals with incomplete spinal cord injury (iSCI) [1–3]. Literature reported that, at 1-year post injury, up to one-third of individuals with recent iSCI would recover partial balance and walking ability [2, 4]. Future ambulatory status is related to the initial amount of motor function below the level of the lesion [5]. For instance, statistics indicate partial recovery of walking function among 80–100% of individuals with iSCI rated D on the American Spinal Injury Association Impairment Scale (AIS), indicating some preservation of motor and sensory function below the level of injury, after the 1st year of injury [2, 6]. This highlights the importance of implementing outcome measures that identify balance and walking capacities of individuals with iSCI to guide the delivery of more effective rehabilitative interventions.

A significant challenge for individuals with iSCI is to maintain postural stability while recovering walking function [7]. iSCI affects the ability to safely stand and perform functional activities in this position [8]. The literature has reported a high occurrence of falling among the SCI population, with up to 78% of these individuals experience at least one fall post-rehabilitation [9–11]. Falls can lead to injuries and hospitalization [9], restriction in community participation [10, 12, 13], and a fear of falling [14]. One of the major factors contributing to falls in this population is the loss of balance [8, 13], highlighting the lack of effective postural control in individuals with iSCI. Furthermore, greater postural control in this population is highly related with a more normal gait pattern, higher stride speed, less reliance on supervision or physical assistance, and more functional ambulatory status [2]. Therefore, the development of fall prevention strategies is associated with effective postural control.

Effective postural control is obtained via the integration of sensory information [7] and the interaction of the body with the changing environment [9]. Due to the sensory and motor impairments at and below the level of the lesion post-SCI [8], sensory reweighting may be affected. This effect on sensory reweighting results from the development of compensatory strategies to maintain postural stability [1, 15]. Consequently, any alteration in the availability of sensory inputs [7, 16] can further challenge postural stability in this population and may lead to a variety of adaptive movement coordination patterns. Hence, identifying the underlying impairments and changes to movement coordination patterns is necessary for effective rehabilitation post-SCI [1, 15].

Observational balance assessment methodologies have been used for balance assessment post-SCI. Yet, they tend to be subjective and provide minor information for understanding the adaptive postural control strategies for compensating balance difficulties [2, 15, 17], highlighting the necessity of a quantitative method to assess postural stability.

Quantitative evaluation of postural stability is usually performed using measures based on the displacement of the center-of-pressure (COP) on a force-platform [8] or using measures based on center-of-mass (COM) acceleration from an inertial measurement unit (IMU) on the lower trunk [18, 19]. Previous studies have used COP-based measures to investigate limits of stability [8] and the effect of sensory information on postural stability [7] post-SCI. The over-reliance on visual cues while walking and standing due to impaired somatosensation was highlighted [7, 16]. Recently, we characterized the effect of distorted visual and somatosensory inputs on postural control using a waist-mounted IMU and compared balance biomarkers between iSCI and able-bodied populations [20].

Due to impaired somatosensation and reduced muscle control, individuals with iSCI may adapt postural movement strategies compared to able-bodied individuals to compensate for reduced postural control. While COP- and COM-based measures are strong indicators of dysfunctional postural control, they do not directly reflect all aspects of the adaptive postural movement strategies employed during standing [21]. Therefore, although such balance biomarkers can indicate reduced postural control post-iSCI, they are unable to reveal the underlying mechanism of how and why the postural control is altered. Measuring the kinematics between the body segments during standing allows us to capture not only the dysfunctional postural control but also how impaired balance is compensated post-iSCI by alteration of inter-segment motions. Kinematic assessment of body segments during standing enables a better understanding of how individuals with iSCI employ adaptive postural strategies to compensate for balance difficulties due to impaired somatosensory feedback. For example, the impaired control on the ankle joint motion for maintaining the body COM stability during standing might be compensated by the altered motion control of the hip joint. During quiet standing, the human body is modeled as single and double inverted pendulums, to study what is known as ankle and hip strategies, respectively. The human body mainly pivots around the ankle joint with increasing contribution of hip motion with larger postural sways. Previous literature [21, 22] has shown that, at sway oscillations below 1 Hz, able-bodied individuals move their trunk and leg in an in-phase manner indicating an ankle strategy. However, at sway oscillations above 1 Hz, trunk and leg motion is anti-phase, indicating a hip or mixed ankle–hip strategy. This implies the domination of the ankle strategy during low-amplitude, low-velocity, or low-frequency motions, whereas the hip strategy dominates during larger sway perturbations [22–24]. Neurological impairments could alter the ankle and hip strategies in affected individuals at different sway frequencies [25]. The selection of segmental coordination pattern (in-phase or anti-phase) and between-patterns transition, may be associated with a loss of stability and pre-selected movement strategy based on the task [21, 22]. Although the balance strategies of able-bodied individuals have been studied in the past, the segmental coordination patterns utilized by the iSCI population during quiet stance are yet to be investigated.

Our recent study [20] showed that individuals with iSCI suffer from reduced stability performance, increased control demand, and a less effective active correction with a higher reliance on visual information and lower reliance on somatosensory information. In the present study, we aim to (1) compare the postural movement between individuals with iSCI and able-bodied individuals to quantify the inter-segment coordination of the trunk and the leg motions; (2) investigate the alteration of postural movement strategies under conditions that challenge balance by affecting somatosensory (standing on hard vs. foam surfaces) and visual (eyes open vs. closed) inputs; and (3) compare test–retest reliability of inter-segment coordination quantification with conventional balance biomarkers for the iSCI population to show that trunk–leg movement coordination has similar test–retest repeatability compared to the traditional balance biomarkers and does not suffer from lack of reliability.

We hypothesized that movement coordination patterns of individuals with iSCI would be affected due to impaired sensory and motor function compared to able-bodied individuals. We also expected that individuals with iSCI would have difficulties adapting trunk–leg movement patterns from the ankle strategy at lower frequencies to mixed strategy at higher frequencies due to their sensory and motor impairment.

## Methods

### Participants

Thirteen individuals with a traumatic or a non-traumatic iSCI and fourteen aged-matched able-bodied individuals voluntarily participated in this study (Table 1). In the present study, we used ± 3 years for age matching. There was no significant difference between the age of the able-bodied participants and individuals with iSCI (p-value = 0.1146). Participants with iSCI were recruited from the outpatient population of the CIUSSS du Centre-Sud-de-l’Île-de-Montréal (Installation Gingras-Lindsay) and the Lyndhurst Centre, Toronto Rehabilitation Institute-University Health Network. The inclusion criteria for iSCI population were: (a) adults with traumatic and non-traumatic motor iSCI with American Spinal Injury Association Impairment Scale (AIS) C or D; (b) at least 5 months post-injury; and (c) able to walk for 6 min without assistive devices or assistance of another person to ensure that intrinsic balance ability could be studied. Exclusion criteria were: (a) presence of other neurological disorders; (b) visual impairments not corrected with glasses; and (c) vestibular deficits. Ethics approval was obtained from the local ethics committees. Each participant provided written informed consent prior to participation.

**Table 1:** Demographic information of participants

	Variable	Mean (standard deviation)	Range
iSCI	Age (years)	52.4 (20.5)	20–87
	Height (cm)	174.7 (7.8)	161–188
	Weight (kg)	82.1 (18.3)	57–113.4
	Time post lesion (months)	62.2 (70.1)	27–289
	Lower extremity motor score (/50)	44.8 (4.3)	32–49
Able-bodied	Age (years)	39.4 (19.3)	18–84
	Height (cm)	170.5 (8.4)	156–181
	Weight (kg)	69.8 (14.4)	47.5–96

	Variable	Number	
iSCI	Sex (male/female)	Male = 12, Female = 1	
	Level of lesion	Paraplegia: 8, Tetraplegia: 5	
	Type of lesion	Traumatic: 10, Non-traumatic: 3	
Able-bodied	Sex (male/female)	Male = 7, Female = 7	

Demographic information of participants with incomplete spinal cord injury (iSCI); and demographic information of able-bodied participants

### Experimental procedure

Participants were asked to perform a one-minute quiet stance with their feet shoulder-width apart under four different sensory conditions: (1) hard surface with eyes open (HS-EO), (2) hard surface with eyes closed (HS-EC), (3) foam surface with eyes open (FS-EO), and (4) foam surface with eyes closed (FS-EC). The purpose of using a foam surface was to alter somatosensory information while standing. Foam pads with medium density and a thickness of 7.62 cm (3 inches) (Velva 60, Domfoam, Canada) were attached to the participants’ shoes using Velcro straps. The eyes closed condition was used to eliminate the effect of visual feedback on balance. Participants were asked to close their eyes for the duration of EC condition. The standing conditions were performed in a randomized order using simple randomization, and rest breaks were taken between trials as needed. The length of the rest breaks was adjusted to eliminate the impact of fatigue on the performance of the participants. Participants with iSCI participated in two testing sessions (2 weeks apart) to assess the test–retest reliability of the proposed outcome measures.

### Data acquisition and human body modeling

**Fig. 1:**
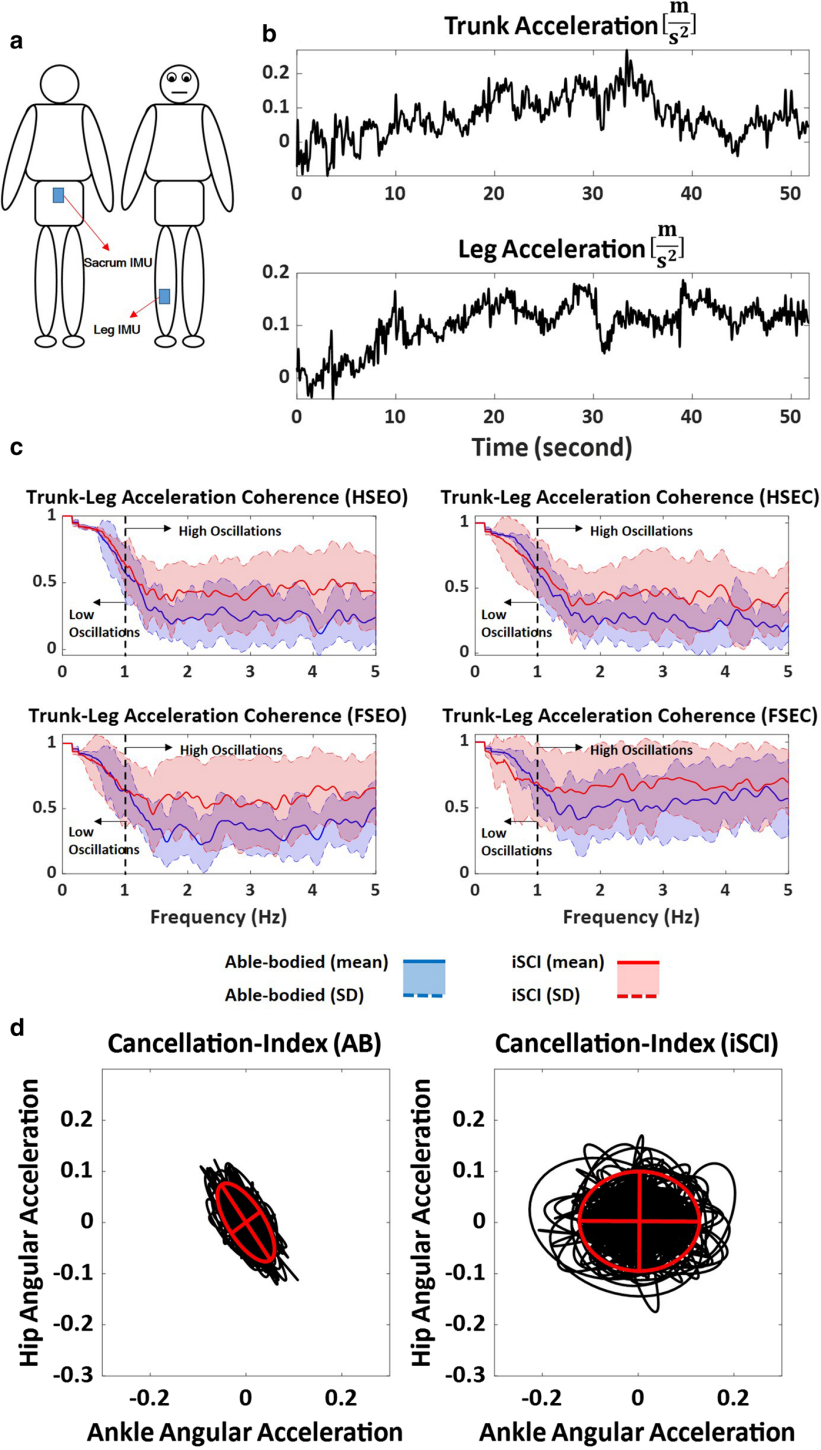
**a** Inertial measurement units (IMUs) were placed on the sacrum and the tibia of the right leg. **b** Acceleration signals in time-domain for trunk and leg segments for one participant for standing on a hard surface with eyes open. **c** Trunk–leg Magnitude-Squared Coherence (MSC) for iSCI population (red) and able-bodied (AB) individuals (blue) presented as an ensemble average (mean ± standard deviation) for both populations and each standing condition on hard surface (HS) and foam surface (FS) with eyes open (EO) and eyes closed (EC). **d** Cancellation-index indicating reciprocal action between the angular acceleration of the ankle and hip joints as presented for one participant from AB and iSCI populations for standing on a hard surface with eyes open

To measure the kinematics of the trunk and leg, we used two IMUs (Physilog^®^5, GaitUp, Switzerland) placed over the sacrum and right tibia of each participant (Fig. 1a, b). Each IMU contained a tri-axial accelerometer (range: ± 16 g) and a tri-axial gyroscope (range: ± 2000 deg/s) and recorded the motion of the body segments at a sampling frequency of 256 Hz. The IMU recordings were low-pass filtered via a zero-lag 8th-order Butterworth filter with a cut-off frequency of 5 Hz.

The human body was modeled as a double inverted pendulum with trunk, leg, and foot segments connected to each other by two 3D revolute joints representing hip and ankle joints. The feet were assumed motionless during the standing trials. The mass, length, COM, and moments of inertia of the segments were estimated based on the body mass and height, according to Winter [26].

We obtained the instantaneous orientation of the trunk and leg segments by aligning the accelerometer’s vertical axis with gravity during quiet stance [27, 28]. We assumed the segments as rigid links and calculated the instantaneous position of the COM, linear acceleration, and angular velocity of the body using the segments’ orientation. We developed a custom-built MATLAB (MathWorks, USA) program for an IMU-based top-down inverse dynamics to estimate the ankle and hip joint moments and center-of-pressure (COP) position based on our previous study [29].

### Outcome measures and data analysis

To identify changes in inter-segment movement coordination and control strategy post-iSCI, we calculated the Magnitude-Squared Coherence (MSC) between the acceleration patterns of the trunk and leg segments in the anterior–posterior direction. MSC was then calculated as:

$$MSC= {{|C}_{xy}\left(f\right)|}^{2}=\frac{{|{P}_{xy}(f)|}^{2}}{{P}_{xx}\left(f\right).{P}_{yy}(f)}$$

where ${C}_{xy}(f)$ and ${P}_{xy}(f)$ are the complex coherence and cross-spectral density between two signals, ${P}_{xx}\left(f\right)$ and ${P}_{yy}\left(f\right)$ are the power spectral densities for the signals being compared, and $f$ is frequency. We calculated the power spectral density and cross-power spectral density using Welch’s averaged method. A Hanning window of 10 s with an overlap of 50% was used across frequencies of 0–5 Hz. The range of the frequency 0 to 5 Hz was selected based on the frequency content of the time-series obtained via Fast Fourier Transform. Previous literature [21, 22] showed that, in able-bodied individuals, trunk and leg have in-phase motions at sway oscillation below 1 Hz, indicating the domination of the ankle strategy during low-frequency motions. However, as the sway oscillation increases above 1 Hz, trunk and leg motions become anti-phase, indicating a hip or mixed ankle–hip strategy during larger sway perturbations [22–24]. Since the literature [21, 22] has shown that a frequency of 1 Hz is the cut-off between in-phase (ankle strategy) and anti-phase (ankle–hip strategy) movement coordination, we calculated the mean of MSC of all frequencies (1) below or equal to 1 Hz, and (2) above 1 Hz for each participant and each standing condition as an outcome measure for balance assessment. An MSC of 1 indicates an in-phase trunk–leg motion pattern, and the smaller the MSC, the lower the degree of in-phase action between trunk and leg segments [22].

We also used the cancellation-index proposed by Kato et al. [30], in addition to MSC, to identify changes in reciprocal action between ankle and hip joints (mixed strategy) during standing post-iSCI as follows:

$$CI= \frac{\sqrt{{k}_{1}^{2}var\left({\ddot{\theta }}_{leg}\right)+{k}_{2}^{2}var\left({\ddot{\theta }}_{trunk}\right)}}{\sqrt{{k}_{1}^{2}var\left({\ddot{\theta }}_{leg}\right)+{k}_{2}^{2}var\left({\ddot{\theta }}_{trunk}\right)+2{k}_{1}{k}_{2}cov({\ddot{\theta }}_{leg},{\ddot{\theta }}_{trunk})}}$$

where $CI$ is cancellation-index, $\ddot{\theta }$ is angular acceleration; ${k}_{1}$ and ${k}_{2}$ are constants obtained based on the mass and length of the segments as explained by Kato et al. [30]; and $var\left(x\right)$ and $cov(x,y)$ represent the variance of $x$ and the covariance of $x$ and $y$, respectively. A cancellation-index of 1 indicates that there is no reciprocal action between ankle and hip joints, and the greater the cancellation-index, the greater the degree of reciprocal action.

To identify changes to movement coordination strategies due to impairment (iSCI vs. able-bodied) and altered sensory inputs (HS vs. FS and EO vs. EC), we performed statistical analyses on MSC-based outcome measures at low and high frequencies. The Kolmogorov–Smirnov test was used to check that the data were normally distributed, followed by the Levene's test to determine the equality of variance. Subsequently, we performed either a three-way Analysis of Variance (ANOVA) or a Kruskal–Wallis test (significance level = 0.05) with Bonferroni correction followed by a multiple comparison post-hoc test (MATLAB 2019b, MathWorks, USA). We also used Cohen’s d effect size to compare the effect of altered sensory inputs on the adaptation of inter-segment coordination between iSCI and able-bodied populations.

Furthermore, we calculated COP-based and COM acceleration-based measures (Table 2) similar to our previous study [20], to compare the test–retest reliability of MSC-based measures with conventional balance biomarkers. We used the intra-class correlation coefficient (ICC) for the model (2, k) to evaluate the reliability of each outcome measure.

**Table 2:** Balance biomarkers

Outcome measure	Nomenclature	Type
Root-mean-square distance	RDIST	COP Time-domain distance measures
Mean distance	MDIST	
Total excursion	TOTEX	
Mean velocity	MVELO	
95% Confidence ellipse area	Area-CE	COP area measure
Sway area	Area-SW	COP Time-domain hybrid measures
Mean frequency	MFREQ	
Median frequency	MEDFREQ	COP Frequency-domain measures
Centroid frequency	CFREQ	
Frequency dispersion	FREQD	
Sway jerkiness	JERK	COM acceleration-based measures
Root-mean-square acceleration	RMS-ACC	
Centroid frequency	CF-ACC	
Cancellation-index	CI	Trunk–leg acceleration pattern coordination
Magnitude-squared coherence	MSC	

As conventional outcome measures, a total of ten center-of-pressure (COP) measures were calculated according to [33]. In addition, three center-of-mass (COM) acceleration-based measures were used based on [19]. For movement coordination, we used cancellation index based on [30] and Magnitude-Squared Coherence (MSC) between trunk and leg segments

## Results

### Effect size between populations

At lower frequencies (f ≤ 1 Hz), mean MSC between trunk and leg accelerations were high (above 0.88 medians across participants) for both able-bodied and iSCI populations across all standing conditions (Table 3a). Moreover, the effect sizes between populations were small, ranging from 0.06 to 0.42. At higher frequencies (f > 1 Hz), mean MSC between trunk and leg accelerations were reduced for both populations. However, at higher frequencies, individuals with iSCI had significantly larger mean MSC between trunk and leg accelerations compared to able-bodied participants with large effect sizes between populations, ranging from 0.53 to 1.13 across all standing conditions.

**Table 3:** Magnitude-squared coherence

(a)	Lower frequencies (f ≤ 1 Hz)			Higher frequencies (f > 1 Hz)		
	AB	iSCI	Cohen’s d	AB	iSCI	Cohen’s d
HS-EO	[0.87, 0.88, 0.89]	[0.86, 0.89, 0.89]	0.34	[0.18, 0.21, 0.29]	[0.29, 0.44, 0.57]	1.13
HS-EC	[0.88, 0.89, 0.9]	[0.84, 0.88, 0.9]	0.36	[0.19, 0.24, 0.33]	[0.27, 0.44, 0.57]	0.99
FS-EO	[0.84, 0.89, 0.9]	[0.85, 0.9, 0.91]	0.06	[0.28, 0.34, 0.47]	[0.43, 0.49, 0.83]	1.11
FS-EC	[0.85, 0.89, 0.9]	[0.78, 0.9, 0.94]	0.42	[0.38, 0.59, 0.72]	[0.52, 0.78, 0.87]	0.53

(b)	Cohen’s d effect size between conditions					
	AB		iSCI			
	f ≤ 1 Hz	f > 1 Hz	f ≤ 1 Hz	f > 1 Hz		
HS-EO vs. HS-EC	0.37	0.08	0.33	0.04		
HS-EO vs. FS-EO	0.02	0.77	0.26	0.58		
HS-EO vs. FS-EC	0.35	1.58	0.41	0.97		
HS-EC vs. FS-EO	0.33	0.79	0.05	0.59		
HS-EC vs. FS-EC	0.01	1.61	0.12	0.96		
FS-EO vs. FS-EC	0.32	1.11	0.16	0.35		

(c)	Cancellation index					
	AB	iSCI	Cohen’s d			
HS-EO	[1.012, 1.018, 1.02]	[1.013, 1.015, 1.018]	0.43			
HS-EC	[1.015, 1.017, 1.019]	[1.012, 1.014, 1.018]	0.58			
FS-EO	[1.013, 1.017, 1.019]	[1.013, 1.016, 1.018]	0.27			
FS-EC	[1.015, 1.016, 1.019]	[1.012, 1.015, 1.018]	0.49			

(a) Mean Magnitude-Squared Coherence (MSC) between trunk and leg accelerations presented as [25%, 50%, 75%] percentiles for able-bodied (AB) participants and individuals with incomplete spinal cord injury (iSCI) at lower and higher frequencies for different standing conditions as well as between-population Cohen’s d effect size. (b) Between-conditions Cohen’s d effect size for AB and iSCI populations at lower and higher frequencies. (c) Cancellation-index proposed by Kato et al. [30] as an indicator of trunk–leg reciprocal action presented as [25%, 50%, 75%] percentiles for AB and iSCI populations with between-population effect size for each standing condition. Cohen’s d effect size was defined as very small (d = 0.01), small (d = 0.20), medium (d = 0.50), large (d = 0.80), very large (d = 1.20), and huge (d = 2.00)

### Effect size between conditions

At lower frequencies, the pairwise comparison between mean MSC at different standing conditions revealed small effect sizes for both populations (Table 3b). However, at higher frequencies, medium and large effect sizes were observed for able-bodied participants ranging from 0.77 to 1.61 showing larger effect sizes with more challenging conditions (Table 3b). Similar patterns were observed for the iSCI population; however, the effect sizes were relatively smaller compared to able-bodied participants at higher frequencies.

### Main effects

The main effect of the health condition (Table 4a) shows no significant differences between able-bodied and iSCI populations for mean MSC of trunk and leg accelerations at lower frequencies (f ≤ 1 Hz). However, at higher frequencies (f > 1 Hz), individuals with iSCI had significantly larger mean MSC between trunk and leg accelerations compared to able-bodied participants (Fig. 1c). Moreover, the cancellation-index was significantly smaller for individuals with iSCI compared to able-bodied participants (Table 4a and Fig. 1d).

**Table 4:** Statistical analysis on Mean Magnitude-Squared Coherence

(a)	Main effects (p-value)					
	iSCI vs. AB	FS vs. HS	EC vs. EO			
MSC (f ≤ 1 Hz)	0.756	0.218	0.564			
MSC (f > 1 Hz)	*0.000*	*0.000*	0.189			
CI	*0.042*	0.995	0.658			

(b)	Interaction effect of surface and vision conditions (p-value)					
	HSEO vs. HSEC	HSEO vs. FSEO	HSEO vs. FSEC	HSEC vs. FSEO	HSEC vs. FSEC	FSEO vs. FSEC
MSC (f ≤ 1 Hz)	0.939	0.727	0.576	0.967	0.896	0.995
MSC (f > 1 Hz)	1.000	0.190	*0.001*	0.214	*0.001*	0.274
CI	0.947	0.995	0.989	0.990	0.996	1.000

(c)	Interaction effect of health and surface conditions (p-value)					
	AB-HS vs. AB-FS	AB-HS vs. iSCI-HS	AB-HS vs. iSCI-FS	AB-FS vs. iSCI-HS	AB-FS vs. iSCI-FS	iSCI-HS vs. iSCI-FS
MSC (f ≤ 1 Hz)	0.956	0.999	0.687	0.910	0.932	0.600
MSC (f > 1 Hz)	*0.003*	*0.006*	*0.000*	0.999	0.134	0.110
CI	0.990	0.301	0.482	0.473	0.673	0.990

(d)	Interaction effect of health and vision conditions (p-value)					
	AB-EO vs. AB-EC	AB-EO vs. iSCI-EO	AB-EO vs. iSCI-EC	AB-EC vs. iSCI-EO	AB-EC vs. iSCI-EC	iSCI-EO vs. iSCI-EC
MSC (f ≤ 1 Hz)	0.920	0.970	0.926	0.998	1.000	0.998
MSC (f > 1 Hz)	0.416	*0.005*	*0.002*	0.270	0.149	0.991
CI	0.999	0.595	0.296	0.680	0.367	0.960

Statistical analysis on Mean Magnitude-Squared Coherence (MSC) between trunk and leg accelerations at lower and higher frequencies and on cancellation-index (CI): (a) the main effect of health (iSCI vs AB), surface (FS vs. HS), and vision (EC vs, EO) conditions; and interaction effect of (b) surface and vision conditions, (c) health and surface conditions, and (d) health and vision conditions. Italic numbers show significant difference (p-value < 0.05)

The main effect of surface condition (Table 4a) revealed a significantly larger mean MSC for standing on FS compared to HS at higher frequencies, while its effect was negligible on mean MSC at lower frequencies and on the cancellation-index. No main effect of vision (EO vs. EC) was observed on the mean MSC and on the cancellation-index.

### Interaction effects

The interaction effect of vision and surface conditions (Table 4b) showed that the FS-EC condition significantly increased mean MSC compared to HS-EO and HS-EC at higher frequencies. In addition, at higher frequencies, mean MSC of able-bodied participants increased while standing on FS compared to HS (Table 4c). Although a similar trend was observed for the iSCI population, its effect was not significant. The effect of EC on mean MSC was not significant for both populations. However, the iSCI population had significantly larger mean MSC even with EO and EC compared to able-bodied standing with EO (Table 4d). At lower frequencies, all interaction effects were not significant for the cancellation-index and mean MSC. The between-population effect sizes for cancellation-index were small to medium ranging from 0.27 to 0.58 for different standing conditions (Table 3c).

### Test–retest reliability

Table 5 shows test–retest reliability as measured via ICC for conventional balance biomarkers, presented in our previous study [20], and mean MSC at lower and higher frequencies for the iSCI population. Among conventional balance biomarkers, only two COP time-domain measures (TOTALX and MVELO) and RMS-ACC showed excellent reliability across all standing conditions. The rest of these measures showed average or poor reliability for the FS-EC or FS-EO conditions. The highest reliability was observed for mean MSC with excellent reliability at all standing conditions.

**Table 5:**
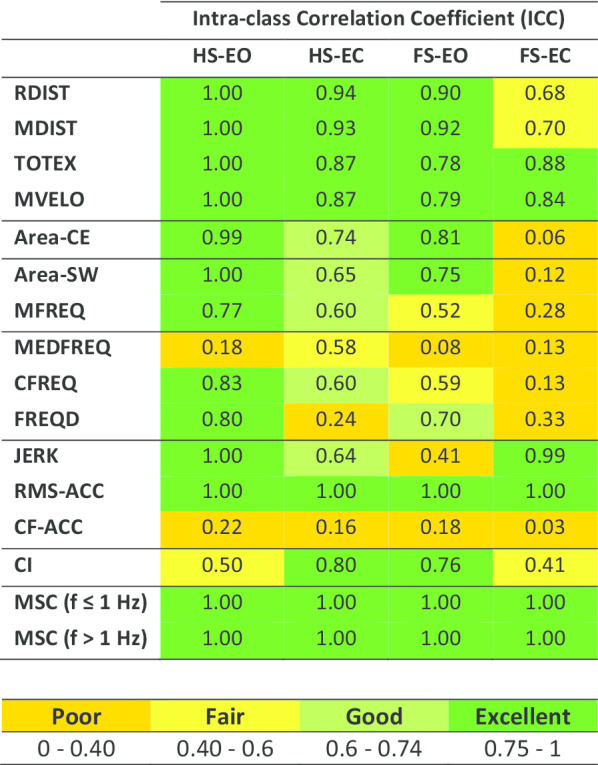
Test–retest reliability

Test–retest reliability of conventional balance biomarkers [20] and Mean Magnitude-Squared Coherence (MSC) between trunk and leg accelerations at lower and higher frequencies for individuals with iSCI as measured by intra-class correlation coefficient (ICC) across different standing conditions on foam (FS) and hard surfaces (HS) with eyes open (EO) and closed (EC)

## Discussion

This study provides a comprehensive evaluation of the balance control strategy and inter-segment movement coordination for individuals with iSCI compared to age-matched able-bodied individuals during a variety of challenging standing conditions that affected somatosensory and visual inputs. Using IMUs placed on trunk and leg, we obtained MSC between the trunk and leg acceleration patterns. We compared mean MSC at lower (f ≤ 1 Hz) and higher (f > 1 Hz) frequencies between populations in different challenging conditions to characterize changes in movement coordination patterns post-iSCI based on reliance on somatosensory and visual information.

Previous studies [21, 22] showed that able-bodied individuals move their trunk and leg in an in-phase motion at sway frequencies below 1 Hz, indicating an ankle strategy. However, at sway frequencies above 1 Hz, the movement of the trunk and leg segments is anti-phase, indicating a hip or mixed ankle–hip strategy. Creath et al. [22] demonstrated that able-bodied individuals have high trunk–leg coherence at lower frequencies, and low trunk–leg coherence at higher frequencies representing ankle (in-phase) and ankle–hip (anti-phase) balance control strategies, respectively. We observed that movement coordination patterns of individuals with iSCI were affected due to impaired sensory and motor function compared to able-bodied individuals. We also observed that individuals with iSCI had difficulties adapting trunk–leg movement patterns from the ankle strategy at lower frequencies to mixed strategy at higher frequencies due to their impaired somatosensation.

### Effect of iSCI on balance strategy

Our results indicate that mean MSC between the trunk and leg acceleration patterns at frequencies below 1 Hz were high (above 0.88 medians across participants) for both groups reflecting an ankle strategy at lower frequencies. No significant main effect of health condition (able-bodied vs. iSCI) was observed on mean MSC at lower frequencies, and we observed small effect sizes between the populations across all standing conditions. These findings imply that the iSCI population display a similar balance control strategy (i.e., ankle strategy) compared to able-bodied individuals at lower frequencies with moving their trunk and leg in an in-phase manner.

As we expected, mean MSC between the trunk and leg acceleration patterns reduced as sway frequency increased from 1.0 to 5.0 Hz in both populations. This highlights the transition from the ankle strategy to the mixed ankle–hip strategy at higher frequencies and is in agreement with previous studies [21, 22]. However, our results revealed that individuals with iSCI showed significantly larger mean MSC at higher frequencies compared to able-bodied participants. Moreover, large effect sizes were observed between the populations in the mean MSC across all standing conditions at higher frequencies. These findings confirm our hypothesis that inter-segment movement coordination is affected post-iSCI due to impaired sensory and motor function compared to able-bodied individuals.

Moreover, as sway frequency increased, able-bodied individuals reduced their trunk–leg acceleration coherence representing a switch from an ankle strategy to a hip or mixed strategy [22]. However, the iSCI population showed a significantly larger mean MSC between trunk and leg accelerations. This indicates that they are less able to adapt their movement patterns from the ankle strategy to a mixed strategy at higher frequencies compared to able-bodied individuals. In addition, we used the cancellation-index from the literature [30, 31] to investigate reciprocal motions of the ankle and hip joints during quiet standing, highlighting the degree of mixed ankle–hip strategy. We observed significantly smaller cancellation-index in the iSCI population compared to able-bodied individuals confirming reduced anti-phase motion between the ankle and hip joints post-iSCI. This also highlights an inability to utilize the mixed ankle–hip strategy for maintaining balance due to impairment in this population.

### Effect of alteration of sensory information

We investigated the effect of altered sensory information on balance control strategy in able-bodied and iSCI populations. We compared mean MSC at lower and higher frequencies under conditions that challenge balance by affecting somatosensory (standing on HS vs. FS) and visual (EO vs. EC) inputs. The main effect of surface condition revealed a significantly larger mean MSC at higher frequencies for standing on FS compared to HS. However, the main effect of surface condition was insignificant on the cancellation-index and the mean MSC at lower frequencies. Larger mean MSC at higher frequencies could imply that when the somatosensory feedback is distorted due to standing on FS, utilizing the mixed ankle–hip strategy is challenged at higher sway frequencies. In contrast, depriving visual information did not reveal any significant effect on the mean MSC at lower and higher frequencies. This highlights the minor effect of vision on the transition from the ankle strategy to ankle–hip strategy at higher frequencies. The interaction effect of surface and vision conditions (Table 4b) revealed a similar finding showing a significant increase in mean MSC at higher frequencies for FS-EC compared to HS-EO and HS-EC conditions while no significant effect of vision was observed.

At higher frequencies, mean MSC significantly increased for able-bodied participants when standing on FS compared to HS. This implies that altered somatosensory information challenged the use of a mixed ankle–hip strategy at higher frequencies for able-bodied individuals. In contrast, the effect of FS compared to HS on mean MSC was insignificant for the iSCI population. This may be associated with impaired somatosensory feedback post-iSCI. iSCI alters somatosensory tracts located in the dorsal column decreasing the relative contribution of somatosensory information to maintaining balance [7] whereas able-bodied individuals primarily use somatosensory information for maintaining balance [7]. This explains why altering somatosensory information significantly affects the balance control strategy in able-bodied individuals while its effect is minor on the iSCI population. Moreover, individuals with iSCI mainly use visual information to maintain postural stability [20] and therefore, altering the somatosensory information by using a foam surface had a lesser impact on the control strategy used by this population.

In agreement with the findings above, the pairwise comparison between mean MSC of different conditions showed small effect sizes at lower frequencies and medium to large effect sizes at higher frequencies for both populations due to alteration of sensory inputs. Between-condition effect sizes were relatively smaller for the iSCI population compared to able-bodied individuals confirming less adaptive movement coordination at higher frequencies post-iSCI.

Note that although the cancellation-index was able to distinguish movement coordination patterns of the iSCI population from able-bodied participants, it was incapable of identifying changes in balance strategies due to altered sensory information, in contrast to MSC. This is due to the fact that the cancellation-index is a time-domain measure that indicates the trunk–leg reciprocal action across the whole frequency spectrum and does not identify the transition from in-phase to anti-phase inter-segment coordination as sway frequency increases. Hence, using the cancellation-index to quantify trunk–leg anti-phase action may not be sensitive enough to identify changes to inter-segment coordination due to the alteration of sensory inputs. In contrast, mean MSC across different ranges of frequency showed sensitivity to alteration of sensory information. This highlights the power of using MSC between trunk and leg accelerations, compared to the cancellation-index, in identifying changes to balance control strategies not only due to neuromuscular impairments but also due to the alteration of sensory inputs.

### Test–retest reliability

Although a majority of the conventional biomarkers of standing balance previously suggested in the literature showed excellent test–retest reliability in the least challenging condition (HS-EO), only three of them (COP Total Excursion, COP Mean Velocity, and COM RMS Acceleration) had good to excellent test–retest reliability in all four conditions. Cancelation index showed good to excellent test–retest reliability in only two conditions. However, MSC in both lower and higher frequencies showed excellent test–retest reliability for all conditions. As such, despite its complex mathematical definition, MSC in both lower and higher frequencies provided repeatable, responsive and sensitive outcome measures with neurophysiological relevance for the evaluation of balance strategy post-iSCI.

### Limitations

The data used in the present study were obtained from a relatively small population of individuals with iSCI, which limits generalization of the observations and reached conclusions. A larger population would be needed to identify any clinically meaningful changes in balance control. Moreover, bilateral symmetry was assumed in this study, which ignores any asymmetric motion patterns between the left and right legs. The assumption of bilateral symmetry for individuals with iSCI was based on our preliminary experimental investigations that did not reflect a significantly weaker (or stronger) side. Participants with iSCI showed relatively good motor recovery and had Lower Extremity Motor Scores (LEMS) of 44.8 ± 4.3 (mean ± standard deviation) out of 50 for both sides. In addition, the LEMS for the right and left sides were 22.6 ± 1.4 and 22.4 ± 3.6, out of 25, respectively, showing no statistical significant difference between the sides (p-value = 0.8336). Nevertheless, this assumption is a limitation of this study.

We assumed the shank and thigh as a single segment, which neglects the relative angle at the knee joint. Literature has shown a minor contribution of the knee joint to the standing balance of able-bodied individuals compared to the ankle and hip joints considered in this study [32]. Despite, the effect of knee joint on the movement coordination of individuals with iSCI during quiet standing should be investigated in the future.

### Future directions

This study highlights how the integration of the sensory inputs and motor strategies are related and how they are impaired following iSCI. It shows the necessity of evaluating sensory integration in individuals with iSCI and observing how the motor control strategies are affected due to iSCI under different sensory conditions. Sensory integration can be evaluated with various devices (such as the Smart Balance Master™) and also with clinical scales such as the mini BESTest. However, most clinical evaluations do not specifically characterize ankle and/or hip strategies. This study suggests that a more comprehensive evaluation of balance in individuals with iSCI should assess how motor control strategies are modified following iSCI. We do not know at this point whether these motor control strategies could be improved if therapists train individuals with iSCI under these various sensory conditions. Nevertheless, therapists can use such an objective method to characterize specific impairments and identify underlying causes. Obtained measures can then be used to precisely focus the therapy on underlying causes and track subtle changes in postural control over time. As a future direction, it could be investigated how trunk–leg movement coordination in the iSCI population would change following rehabilitative interventions. In addition, it could provide clinicians with an insight into how adaptive movement strategies affect postural control post-iSCI.

## Conclusion

We presented a comprehensive assessment of balance control strategy and inter-segment movement coordination for the iSCI population compared to age-matched able-bodied participants during standing on hard and foam surfaces with eyes open and closed using only two IMUs. We observed a similar balance strategy at lower frequencies between iSCI and able-bodied populations. However, we observed a decreased ability post-iSCI in adapting inter-segment coordination between trunk and leg segments changing from ankle strategy to mixed ankle–hip strategy as the sway frequency increases. Using coherence between trunk and leg accelerations, we also showed that alteration of somatosensory inputs could affect trunk–leg movement coordination in both populations. Characterization of trunk–leg movement coordination based on coherence analysis provided a sufficient sensitivity with discriminatory ability and excellent test–retest reliability to identify changes in balance control strategy post-iSCI. Conventional IMU-based balance biomarkers were not able to obtain a similar extent of responsiveness and repeatability. Our proposed method could be used in the future for objective outcome evaluation of rehabilitative interventions on postural control post-iSCI.

## Data Availability

The datasets used and analyzed during the current study are available from the corresponding author on reasonable request and with permissions of the Research Ethics Board of the University of Alberta, and the Research Ethics Board of the University Health Network.

## Abbreviations

AB: Able-bodied
iSCI: Incomplete spinal cord injury
AIS: American Spinal Cord Injury Association Impairment Scale
COP: Center of pressure
COM: Center of mass
IMU: Inertial measurement unit
HS: Hard surface
FS: Foam surface
EO: Eyes open
EC: Eyes closed
MSC: Magnitude-squared coherence
ANOVA: Analysis of variance
ICC: Intraclass correlation coefficient
